# The Effect of Osmotic Dehydration Conditions on the Magnesium Content in Beetroot (*Beta vulgaris* L.)

**DOI:** 10.3390/molecules30143051

**Published:** 2025-07-21

**Authors:** Bartosz Kulczyński, Joanna Suliburska, Anna Gramza-Michałowska, Andrzej Sidor, Przemysław Łukasz Kowalczewski, Anna Brzozowska

**Affiliations:** 1 Department of Gastronomy Science and Functional Foods, Faculty of Food Science and Nutrition, Poznań University of Life Sciences, Wojska Polskiego 31, 60-624 Poznań, Poland; anna.gramza@up.poznan.pl (A.G.-M.); andrzej.sidor@up.poznan.pl (A.S.); anna.brzozowska@up.poznan.pl (A.B.); 2 Department of Human Nutrition and Dietetics, Faculty of Food Science and Nutrition, Poznań University of Life Sciences, Wojska Polskiego 31, 60-624 Poznań, Poland; joanna.suliburska@up.poznan.pl; 3 Department of Food Technology of Plant Origin, Faculty of Food Science and Nutrition, Poznań University of Life Sciences, Wojska Polskiego 31, 60-624 Poznań, Poland; przemyslaw.kowalczewski@up.poznan.pl; 4 Collegium Medicum, Andrzej Frycz Modrzewski Krakow University, 30-705 Kraków, Poland

**Keywords:** beetroot, food fortification, osmotic dehydration, magnesium, inulin, antioxidant activity

## Abstract

Osmotic dehydration is a process involving a two-way mass transfer, during which water and substances dissolved in it are removed from the product and, at the same time, substances dissolved in a hypertonic solution penetrate into the tissues. This process has a significant effect on, among other things, the nutritional and sensory parameters, as well as the texture and shelf life of the dehydrated product. This study analyzed the effect of osmotic dehydration of beet on magnesium content following the addition of various chemical forms of magnesium (magnesium oxide, magnesium citrate, magnesium chloride) to a hypertonic solution. Magnesium was added in concentrations of 2.5 or 5.0% relative to the mass of the solution. The following compounds were used to prepare hypertonic solutions (25 and 50%): inulin, xylitol, erythritol, and sucrose. The control sample was water. A significant increase in magnesium content in the dehydrated material was confirmed. This effect was determined by many factors, among which the most important were the chemical form of magnesium, the type of osmotically active substance, magnesium concentration, and process time. The highest magnesium content was found in samples dehydrated in a 50% inulin solution with a 5.0% addition of magnesium chloride under the following conditions: 120 min/30 °C. It was also demonstrated that osmotically dehydrated samples exhibited approximately 3–5 times lower antioxidant activity in DPPH, ABTS, and ORAC tests.

## 1. Introduction

In recent years, there has been a growing focus on the development of functional foods, i.e., food products that, in addition to their basic nutritional value, provide additional health benefits and help prevent chronic diseases. This trend reflects growing awareness of the impact of diet on well-being and the need for innovative solutions to address widespread nutrient deficiencies. One such deficiency of global concern is magnesium.

The literature indicates that 60% of adults do not achieve the average dietary intake (ADI) of magnesium. Moreover, as many as 45% of the population may be deficient in this mineral. Considering the fact that magnesium is one of the most important minerals in the human body (it is involved in approx. 80% of metabolic processes) [1], its deficiency is associated with many health problems. Studies confirm that magnesium deficiency is associated with a higher risk of developing high blood pressure [2], coronary heart disease [3], atrial fibrillation [4], type 2 diabetes [5], Alzheimer’s disease [6], and depression [7]. In addition, magnesium deficiency reduces the quality of sleep [8], increases the risk of bone fractures [9], and predisposes to the development of chronic low-grade inflammation [10]. Researchers emphasize that there has been a significant reduction in the level of magnesium in the soil over the past few decades. This results in a lower magnesium content in plants, which are the main dietary source of this mineral in the human diet [11,12]. Therefore, it is not only unhealthy eating habits that are responsible for the low magnesium intake. For this reason, it is even more important to find and develop solutions that provide consumers with magnesium-rich food.

One promising approach is to enrich fruits and vegetables—natural carriers of bioactive compounds—with minerals and other nutrients. In this context, physicochemical techniques such as osmotic dehydration have attracted interest [13,14]. Osmotic dehydration is a process of removing water from biological materials by osmosis, utilizing the difference in osmotic pressure between the interior of cells and the hypertonic solution in which they are immersed. As a result, water migrates through semi-permeable membranes to the outside of the cells, while some of the substances dissolved in the solution penetrate into the tissue. This process plays an important role in the food industry, especially in the context of food preservation, modification of its sensory properties, and improvement of its health benefits [15,16,17].

The efficiency of osmotic dehydration depends on several key factors: (1) the type of osmotic solution—sucrose and sodium chloride are most commonly used, although inulin, polyols (e.g., xylitol, erythritol, maltitol, sorbitol) and fruit juice concentrates are increasingly being used, which also provide bioactive ingredients; (2) the concentration of osmotically active substances—the higher the concentration of the solution, the greater the osmotic pressure and the faster the dehydration process; (3) the process time and temperature—higher temperatures increase the rate of water diffusion but can also lead to the degradation of bioactive ingredients. The type and structure of plant tissue and the addition of other substances to the osmotic solution are also important [18,19,20].

In this study, the effect of different osmotic parameters on the magnesium content in beetroot was analyzed. For this purpose, different chemical forms of magnesium (magnesium oxide, magnesium citrate, and magnesium chloride) were used. Magnesium compounds were added in two concentrations (2.5% and 5.0%) to a hypertonic solution prepared using inulin, xylitol, erythritol, and sucrose. It is worth noting that the osmotic substances used, such as inulin and erythritol, have proven broad health benefits. Studies have shown that inulin has prebiotic properties and increases the number of bifidobacteria in the intestines [21]. Furthermore, inulin promotes the absorption of minerals from the intestine (magnesium, zinc, copper, calcium) [22,23,24], lowers the fasting blood glucose level [25], lowers triglyceride levels [26], reduces blood pressure [27], has anti-inflammatory properties [28], and lowers the blood uric acid level [29]. Erythritol, on the other hand, inhibits the growth of bacteria that cause tooth decay [30]. Furthermore, adding it to products (e.g., drinks) increases satiety, which can be important for weight loss [31].

In the context of functional food production, the use of osmotic dehydration to enrich plants with selected nutrients is of particular interest. To date, a wide variety of vegetables and fruits have been subjected to osmotic dehydration, including mango [32], kumquat [33], pineapple [34], apple [35,36], strawberry [37], kiwi [38], banana [39], papaya [40], and carrot [41]. Pumpkin and beetroot were used in previous research [42,43]. However, it should be emphasized that other research has mainly focused on the impact of osmotic dehydration on the kinetics of solid mass gain and water loss, as well as on the retention of bioactive components present in plants and antioxidant potential. However, there is still a lack of research analyzing the effect of osmotic dehydration on the enrichment of the plant matrix with nutrients. Nevertheless, some studies confirm that osmotic dehydration can be an effective method of increasing the nutritional value of food. For example, Silva et al. showed that the addition of calcium lactate to the hypertonic solution caused a significant increase in the calcium content of osmotically dehydrated pineapple [44]. A similar effect was observed in studies conducted with guava and apples [45,46]. Other studies have confirmed the use of osmotic dehydration to enrich pineapple with probiotic bacteria (*Lactiplantibacillus plantarum*, *Lacticaseibacillus casei*) [47] and to enrich mango with vitamin C [48]. Similarly, recently published research has confirmed the usefulness of osmotic dehydration for enriching plant-based raw materials with nutrients. For example, it was shown that the addition of calcium carbonate (5%) to a 50% solution of inulin or xylitol increased the calcium content in pumpkin to 1328.4 mg/100 g and 1380.4 mg/100 g, when osmotic dehydration was carried out at 50 °C for 2 h. At the same time, it was confirmed that the effectiveness of the increase in calcium content during osmotic dehydration is influenced by process parameters, including temperature and duration, type and concentration of osmotic substance, chemical form of calcium, and calcium concentration [43]. Another own research analyzed the influence of osmotic dehydration on the potassium content in beets. It was observed that among the three chemical forms of potassium (potassium gluconate, potassium citrate, potassium chloride), the highest potassium content in osmotically dehydrated beets was obtained when potassium chloride was used. The potassium content of the beets was 5779.03 mg/100 g and 5457.28 mg/100 g for the samples dehydrated in a 50% solution of erythritol and inulin for 180 min [42].

Additionally, the effect of osmotic dehydration of beetroot on its antioxidant activity was examined in the following tests: ABTS, DPPH, and ORAC, and a texture profile analysis of osmotically dehydrated samples was performed.

## 2. Results and Discussion

### 2.1. Magnesium Content in Dehydrated Beetroot Flesh (First Stage)

**Figure 1:**
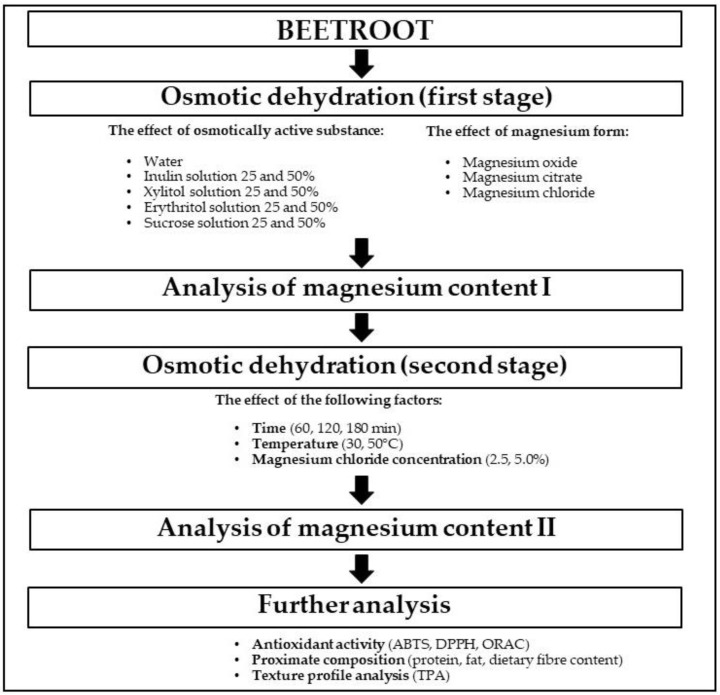
Research scheme.

The research part concerning the influence of osmotic dehydration on magnesium content was divided into two stages (Figure 1).

In the first stage of the study, the influence of the following factors on the magnesium content in beet subjected to osmotic dehydration was analyzed: type of osmotic substance, concentration of hypertonic solution, and chemical form of magnesium. Inulin, erythritol, xylitol, and sucrose were selected as osmotic substances. Solutions of these substances were used at concentrations of 25 and 50%. Three chemical forms of magnesium were used: magnesium oxide, magnesium citrate, and magnesium chloride, in an amount of 5.0% relative to the weight of the hypertonic solution. The highest magnesium content was observed in the samples dehydrated with magnesium chloride (327.36–684.96 mg/100 g), which may be related to the very high solubility of this compound in water (2350 g/L (20 °C)) (Table 1). For comparison, the magnesium content in raw beetroot that had not undergone osmotic dehydration was 16 mg/100 g.

In contrast, the lowest level of magnesium was found in samples with the addition of magnesium oxide (303.25–585.52 mg/100 g), which has a very low solubility in water (6.2 mg/L (20 °C)). It can be seen that the obtained result is more influenced by the solubility of magnesium compounds than by the percentage of magnesium in the molecule (magnesium chloride hexahydrate: 11.96%; magnesium oxide: 60.3%; magnesium citrate: 16.17%). Similarly, compared to the samples dehydrated with magnesium oxide, the samples dehydrated with magnesium citrate, which has a higher solubility in water (20 g/100 mL (20 °C)), had a higher magnesium content (335.75–612.05 mg/100 g).

Among the analyzed types of osmotic solutions, the highest magnesium content was observed in samples dehydrated in inulin and erythritol solutions. A slightly lower magnesium content was observed in samples dehydrated in xylitol and sucrose solutions (inulin solution: 303.25-683.96 mg/100 g; erythritol solution: 401.72–668.38 mg/100 g; xylitol solution: 255.90–604.78 mg/100 g; sucrose solution: 282.19–589.81 mg/100 g).

As expected, all samples that were dehydrated in more concentrated solutions of osmotically active substances (50% vs. 25%) had a higher magnesium content. An increase in the concentration of a substance in an osmotic solution causes an increase in its osmotic pressure. As a result, larger amounts of the components dissolved in the solution are transferred into the sample being dehydrated. Furthermore, the higher the concentration of the osmotic solution, the more water is removed from the plant matrix cells. This, in turn, causes more intensive dehydration of the sample and concentration of the ingredients it contains. The same observations were made in previous studies, which analyzed the effect of osmotic dehydration on the calcium content in pumpkin and the potassium content in beets [42,43].

Among all the analyzed samples, the highest magnesium content was found in samples dehydrated in a 50% inulin solution with magnesium chloride (683.96 mg/100 g) and samples placed in a 50% erythritol solution, also with the addition of magnesium chloride (668.38 mg/100 g). There were no statistically significant differences between these samples (*p* > 0.05). As expected, the magnesium level was lowest in the samples placed in water (87.31–144.81 mg/100 g). At the same time, it should be noted that multivariate analysis of variance proved that the effect obtained was equally influenced by both the chemical form of magnesium and the type of osmotically active substance (η^2^: 0.98 vs. 1.00) (Table 2). Similarly, the same effect was observed in our previous work, which indicated that the potassium content in the dehydrated beet was dependent on the type of osmotically active substance (η^2^: 0.99) and the chemical form of potassium (η^2^: 1.00) [42].

In turn, Table 3 shows the magnesium content in osmotically dehydrated beets under the same conditions, which were additionally freeze-dried. The highest magnesium content was found in the sample dehydrated in a 50% sucrose solution with the addition of magnesium oxide (2260.42 mg/100 g of lyophilisate) and in the sample immersed in water with the addition of magnesium chloride (2030.05 mg/100 g of lyophilisate). The obtained result was determined by the dry matter content.

### 2.2. Magnesium Content in Dehydrated Beetroot Flesh (Second Stage)

In the second stage of the research, the effect of the following osmotic dehydration parameters on the magnesium content in beet was analyzed: temperature (30 and 50 °C), time (60, 120, 180 min), and magnesium concentration (2.5 and 5.0%). Based on the results obtained in the first stage of the research, a 50% solution of inulin and erythritol was selected as the osmotic solution. Magnesium chloride was selected as the magnesium form (Table 4).

As expected, the dehydration samples with a 5% magnesium chloride additive had a statistically significantly higher magnesium content (*p* < 0.05). This effect was observed in all samples. Similar observations were made in previous studies comparing the addition of calcium carbonate (2.5% vs. 5.0%) and potassium chloride (2.5% vs. 5.0%) [42,43]. Furthermore, it was noted that the duration of osmotic dehydration has a significant impact on the magnesium content. It was shown that the longer the process time, the higher the magnesium content in the dehydrated samples, which is also consistent with previous studies. At the same time, it was confirmed that the process temperature also affects the result obtained. For nine pairs of samples, a higher magnesium content was observed when the osmotic dehydration temperature was 50 °C. For the remaining three pairs of samples, the opposite effect was found—samples dehydrated at 30 °C were characterized by a higher magnesium content. Although statistically significant differences were found for most of the pairs of samples (*p* < 0.05), this effect was not as noticeable as in the case of the other parameters (processing time and magnesium concentration).

Results of multivariate analysis of variance for dehydrated beetroot samples confirm that the most significant impact on the effectiveness of osmotic dehydration in terms of increasing the magnesium level in beetroot was exerted by magnesium concentration (η^2^ = 1.00) and process time (η^2^ = 0.99) (Table 5). On the other hand, the impact of process temperature was significantly lower (η^2^ = 0.45). Similar conclusions were noted in previous studies, where process time (η^2^ = 0.99) and potassium concentration (η^2^ = 0.99) also had the most significant impact on the obtained result, while process temperature had the least impact (η^2^ = 0.54) [42].

The highest magnesium content was found in samples obtained under the following osmotic dehydration conditions: (1) 50% erythritol solution, 5.0% magnesium addition, 180 min, 50 °C (678.91 mg/100 g), (2) 50% inulin solution, 5.0% magnesium additive, 180 min, 30 °C (672.45 mg/100 g), (3) 50% inulin solution, 5.0% magnesium additive, 120 min, 50 °C (664.59 mg/100 g), and (4) 50% erythritol solution, 5.0% magnesium addition, 180 min, 30 °C (663.31 mg/100 g). In contrast, the lowest magnesium content was found in dehydrated samples with a 2.5% addition of magnesium chloride in 50% erythritol solutions (60 min, 30/50 °C) (156.57–178.85 mg/100 g) and 50% inulin solutions (60 min, 30/50 °C) (181.60–194.93 mg/100 g).

In the case of the samples with the highest magnesium content, it would be sufficient to consume only 25 g of dehydrated beetroot enriched with magnesium to cover the Recommended Dietary Allowances by ~40/50% (men/women). This confirms that osmotic dehydration is an effective method of enriching the plant matrix with magnesium and that such enriched foods can be an important addition to a diet lacking in magnesium.

Based on the results obtained, the level of magnesium absorption (%) in the analyzed samples of osmotic dehydrated beetroot was calculated (Table 6).

**Figure 2:**
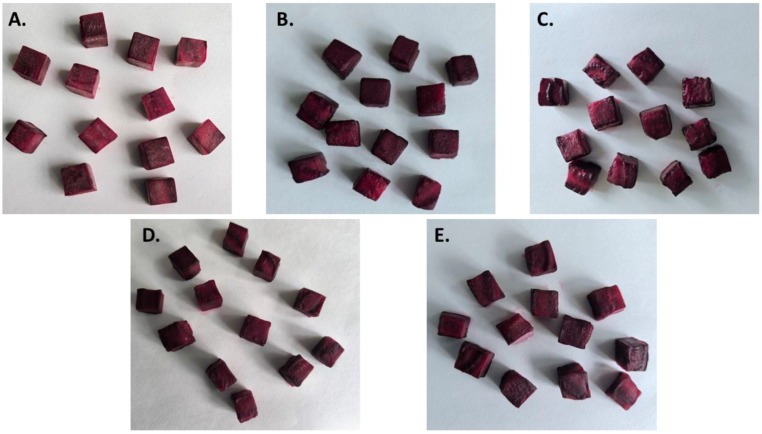
Pictures of osmotically dehydrated beetroot. (**A**)—raw, fresh beetroot; (**B**)—beet subjected to osmotic dehydration (50% inulin solution, 2.5% magnesium chloride); (**C**)—beet subjected to osmotic dehydration (50% inulin solution, 5.0% magnesium chloride); (**D**)—beet subjected to osmotic dehydration (50% erythritol solution, 2.5% magnesium chloride); (**E**)—beet subjected to osmotic dehydration (50% erythritol solution, 5.0% magnesium chloride).

Depending on the parameters, the magnesium absorption ranged from 10.47 to 23.81%. A higher level of absorption was found for samples dehydrated for longer times (120 and 180 min). In all cases, statistically significant differences (*p* < 0.05) were found between samples dehydrated in solutions with different magnesium concentrations (2.5% and 5.0%). However, in the case of 8 pairs of samples, a higher absorption of magnesium was found when 5.0% magnesium chloride was used, while in the case of the remaining four pairs of samples, the result was higher when 2.5% magnesium chloride was used. The temperature of the process also influenced the level of magnesium absorption. Statistically significant differences (*p* < 0.05) were observed in eight pairs of samples, while no significant differences in the magnesium absorption level were found for the remaining four pairs of samples (*p* > 0.05). In most cases, samples dehydrated at a higher temperature (50 vs. 30 °C) were characterized by a higher level of magnesium absorption. Pictures of osmotically dehydrated beetroot are shown in Figure 2A–E.

In comparison to previous studies, in which pumpkin flesh was enriched with calcium by osmotic dehydration, a higher level of mineral absorption was noted. In the case of pumpkin enrichment, the calcium absorption level was between 6.22% and 14.94%. This difference in results could have been caused mainly by the form of calcium used in the research—calcium carbonate, which has a very low solubility in water. The magnesium content of the osmotically dehydrated and freeze-dried samples ranged from 284.09 to 1590.68 mg/100 g (Table 7). As expected, the highest magnesium content was found in the samples dehydrated with 5.0% magnesium.

Although osmotic dehydration is commonly used in research as a method for shaping various product characteristics, including sensory attributes, shelf-life, water activity, kinetics of water loss, and antioxidant potential, few studies have been conducted to date with the main objective of enriching plant materials with minerals and other compounds. As mentioned above, previous own research has confirmed the effectiveness of osmotic dehydration to enrich pumpkins with calcium and beets with potassium [42,43].

For example, it has been shown that the use of the following osmotic dehydration parameters: 50% erythritol solution, 30 °C, 180 min, 5.0% potassium chloride addition resulted in a significant increase in the potassium content in beets (5779.03 mg/100 g). In turn, significantly lower potassium content was observed in samples dehydrated with the addition of other forms of potassium: potassium gluconate (1301.28–1934.5 mg/100 g) and potassium citrate (2433.94–4515.05 mg/100 g) [42]. When pumpkin was dehydrated, the highest calcium content was found in the sample with added calcium carbonate (188.5–1409.0 mg/100 g). Lower levels were observed in samples dehydrated with calcium lactate (438.7–951.9 mg/100 g) and calcium chloride (809.8–1395.1 mg/100 g) [43].

Other authors have shown that osmotic dehydration, for example, can be a useful method for enriching oranges with vitamin C [49]. In this experiment, various hypertonic solutions were used, including strawberry juice concentrate and rosehip juice as a natural source of vitamin C. Unlike in our study, no enrichment ingredient was added to the hypertonic solution. Galus et al. proved that dehydrated samples in strawberry juice concentrate and rosehip juice had a statistically significantly higher vitamin C content (62.32 and 80.27 mg/100 g) compared to fresh-cut oranges (42.73 mg/100 g). However, the authors of the experiment did not analyze the influence of other process parameters, such as time and temperature. The addition of ascorbic acid to hypertonic solutions was used in research conducted by Nagai et al. [48]. The scientists used 40% and 60% sucrose solutions to which they added ascorbic acid in concentrations ranging from 0.5 to 2.0 g/100 g of solution. They showed that the addition of ascorbic acid increased the vitamin C content of the mango from 40.28 to as much as 400.53 mg/100 g of fruit.

However, it should be noted that osmotic dehydration using solutions that are not also a source of bioactive compounds reduces the level of these components in the product. For example, Wojtyś et al. [50] demonstrated that osmotic dehydration of Japanese quince fruits in erythritol or sucrose solutions resulted in a statistically significant (*p* < 0.05) reduction in vitamin C content (from 4.81 to 0.67–1.25 mg/g dm.).

The effect of different osmotic dehydration conditions on calcium gain in chilacayote (*Cucurbita ficifolia* Bouché) was the subject of research conducted by Rodríguez-Ramírez et al. [51]. They confirmed that calcium content is influenced by temperature and process time. It was shown that the higher the temperature of osmotic dehydration (20 °C vs. 35 °C vs. 50 °C), the higher the calcium content in the product. At the same time, they found that samples dehydrated over a longer time (4.5 h) were characterized by a higher calcium content compared to samples dehydrated for a shorter time (1.5 and 3.0 h). The authors of the experiment noted a calcium gain ranging from 11.8 to 46.8 mg per 100 g of fresh sample. In this study, calcium hydroxide (Ca(OH)_2_) was the source of calcium.

As Lech et al. [52] have shown, osmotic dehydration can also be a technique for enriching the plant matrix with polyphenols. The researchers observed that the use of chokeberry juice as a hypertonic solution resulted in a statistically significant increase in the total polyphenol level in the dehydrated carrots and zucchini. At the same time, the authors of the experiment confirmed that samples dehydrated for a longer time (100–120 min) had a higher content of these compounds, compared to samples dehydrated for 20, 40, and 60 min, which confirms that time is an important parameter determining the effectiveness of osmotic dehydration. For example, in carrots subjected to osmotic dehydration for 2 h, an increase in total polyphenol content was observed from approx. 130 mg/100 g dm. to approx. 1100 mg/100 g dm.

Similarly, Nićetin et al. [53] confirmed that osmotic dehydration can be an effective method of enriching plant materials with phenolic compounds. As a hypertonic solution, they used sugar beet molasses, which is a natural source of bioactive compounds. The researchers showed that osmotic dehydration of celery in beet molasses increased the content of phenolic compounds such as apigenin, luteolin, chlorogenic acid, kaempferol, gallic acid, and chrysin. Especially high efficiency of enriching celery with phenolic compounds was obtained when the dehydration process lasted >3 h 35 min. The highest content of these ingredients was achieved during 5 h 50 min of osmotic dehydration.

Based on research to date, it can be confirmed that osmotic dehydration is an effective method of enriching food products with specific nutrients, including vitamins, minerals and antioxidants.

### 2.3. Antioxidant Activity and Proximate Composition of Dehydrated Beetroot

Osmosis dehydration is a process that affects the antioxidant activity of food. Studies have shown that products subjected to osmosis dehydration have lower antioxidant properties compared to fresh raw materials.

**Figure 3:**
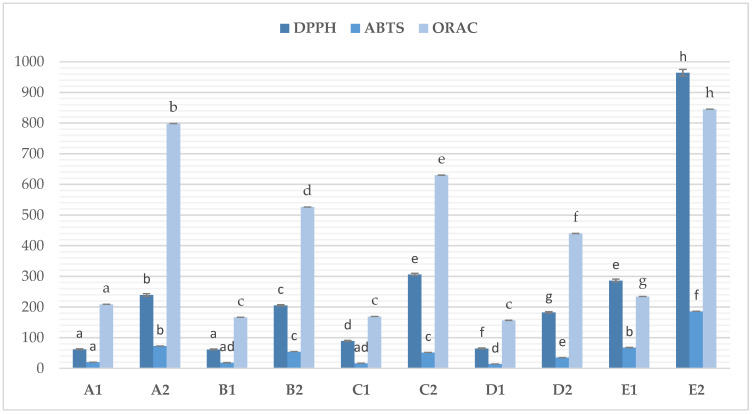
Antioxidant activity of osmotic dehydrated beetroot. Sample A1/A2—beet subjected to osmotic dehydration (50% inulin solution, 5.0% potassium chlo-ride addition, 180 min, 30 °C, before and after freeze-drying; Sample B1/B2—beet subjected to osmotic dehydration (50% inulin solution, 5.0% potassium chloride addition, 180 min, 50 °C, before and after freeze-drying; Sample C1/C2—osmotically dehydrated beet (50% erythritol solution, 5.0% potassium chloride addition, 180 min, 30 °C, before and after freeze-drying; Sample D1/D2—osmotically dehydrated beet (50% inulin solution, 5.0% potassium chloride addition, 180 min, 50 °C, before and after freeze-drying; Sample E1—fresh beet; Sample E2—freeze-dried beet. ^a–h^—different letters mean statistically significant differences (*p* < 0.05) between the effect of osmotic dehydration on the antioxidant activity of beetroot.

The conducted research confirmed that osmotic dehydration has a significant effect on antioxidant activity (Figure 3).

The DPPH assay showed that dehydrated samples were characterized by statistically significant (*p* < 0.05) lower antioxidant activity compared to fresh beetroot that had not undergone this process. This activity was found to be approximately 3–5 times lower (182.54–306.22 vs. 964.39 g mg Trolox/100). A similar effect was also observed in ABTS (35.19–73.04 vs. 186.05 mg Trolox/100 g) and ORAC tests (439.91–798.42 vs. 845.43). This effect is caused by the loss of bioactive components as a result of their transfer to the hypertonic solution and the process temperature, although for most of the analyzed samples, the process temperature did not have a significant effect (*p* > 0.05). This could be related to the small difference in osmotic dehydration temperatures (30 vs. 50 °C).

Similarly, an experiment conducted by Galus et al. [49] showed that osmotic dehydration of fresh-cut oranges reduced the ability of these fruits to scavenge the ABTS radical cation. A reduction in antioxidant activity from 7.7 mg TE/d.m. to 2.7–2.8 mg TE/d.m. was observed in samples dehydrated in sucrose and xylitol solutions. However, it should be noted that the effect of osmotic dehydration on antioxidant activity is determined by the type of osmotic substance used. For example, the same authors reported an increase in the antioxidant activity of oranges when they were dehydrated using cherry concentrates (8.5 mg TE/d.m.), rosehip juice (8.8 mg TE/d.m.) and strawberry juice (11.0 mg TE/d.m.), which are natural sources of bioactive compounds with high antioxidant potential.

These observations were confirmed by Wojtyś et al. [50]. The researchers proved that osmotic dehydration caused a decrease in the antioxidant activity of Japanese quince in DPPH (from 5.54 to 1.25–2.27 µM TE/g d.m.) and ABTS (from 14.71 to 4.38–5.96 µM TE/g d.m.) tests. As the scientists proved, the decrease in antioxidant potential was associated with a statistically significant (*p* < 0.05) reduction in the content of vitamin C, total flavonoids, and total phenolics. It should be noted that the following hypertonic solutions were used in this experiment: erythritol 30 and 40% and sucrose 50%.

Similar conclusions were drawn by Yazidi et al. [54], who osmotically dehydrated orange fruits in sucrose and prickly pear molasses solutions. They noted a significant reduction in the ability of dehydrated oranges to scavenge ABTS radical cations (from 7.73 to 2.68 mg Trolox/g d.m.) when sucrose solution was used. At the same time, a decrease in antioxidant potential was observed in samples dehydrated with prickly pear molasses solution, although this reduction was not as significant (6.26 mg Trolox/g d.m.) as in the case of sucrose solution. This effect was caused by some antioxidant activity of the molasses solution itself (3.50 mg Trolox/g w.m.).

Previous studies have also demonstrated that osmotic dehydration has an adverse effect on the antioxidant activity of food. Depending on the hypertonic solution used (50% inulin or erythritol solution) and the process temperature (30 or 50 °C), a decrease in the antioxidant potential of beetroot was observed both in the ABTS (from 63.24 to 21.42–36.85 mg Trolox/100 g) as well as in the DPPH test (from 242.07 to 75.65–109.77 Trolox/100 g) [42].

At the same time, it should be noted that the duration of osmotic dehydration also affects the antioxidant properties of food products. Although this relationship was not analyzed in this study, other studies clearly demonstrate that such a relationship exists. For example, Devic et al. [55] demonstrated that prolonging the osmotic dehydration of apples (from 30 to 180 min) causes a statistically significant increase in the loss of bioactive compounds such as hydroxycinnamic acids, monomeric catechins, and procyanidins in apples, which determine the antioxidant activity of these fruits. Similarly, Rahman et al. [56] observed that the longer the osmotic dehydration time, the lower the antioxidant activity of nutmeg pericarp. Furthermore, the researchers noted higher antioxidant potential in more concentrated hypertonic solutions (70 and 80%). The lowest ability to scavenge DPPH radicals was observed in samples dehydrated in a 60% sugar solution. This effect is likely due to the fact that at high concentrations of the osmotic solution, a layer of osmotic substance forms on the surface of the raw material, which inhibits the loss of low molecular weight antioxidant compounds.

However, it should be noted that the relationship between the concentration of osmotic substances and the antioxidant activity of the dehydrated product requires further investigation, as the observations are not conclusive. For example, Wiktor et al. [57] observed that strawberries dehydrated in a 30% mannitol solution had a statistically significantly higher ability to scavenge ABTS radical cations compared to samples dehydrated in a 20% mannitol solution. However, the opposite effect was observed with sorbitol solutions. Strawberries placed in a 20% sorbitol solution had significantly higher antioxidant potential compared to samples dehydrated with 30% and 40% sorbitol solutions. The authors of the experiment indicated that this effect may be related to the plasticization of cell biopolymers, which may affect the mobility of water and water-soluble compounds.

Although, as has been shown, osmotic dehydration can be used to enrich foods with specific nutrients, it should be noted that it can also have a negative effect on the antioxidant potential of foods. This is important because many studies have shown that Dietary Total Antioxidant Capacity has a significant beneficial effect on human health [58,59,60]. For this reason, it is important to take into account factors that would preserve or even increase the antioxidant potential of dehydrated food when designing osmotic dehydration conditions. As demonstrated by the researchers cited above, this can be achieved by using appropriate hypertonic solutions (e.g., juices, concentrates, and fruit molasses). Another solution may be to add spices with high antioxidant activity to hypertonic solutions, including cloves, oregano, rosemary, thyme, and cinnamon. These ingredients should not only have a positive effect on the antioxidant potential of food, but can also influence the sensory properties of the final product. Furthermore, in the next studies on osmotic dehydration, the use of fruit pomace (raspberry, chokeberry, strawberry, blackcurrant, and elderberry) rich in polyphenols is planned. The proximate composition of dehydrated beetroot is shown in Table 8.

### 2.4. Texture Profile Analysis (TPA)

Osmotically dehydrated samples were subjected to texture profile analysis, and the following parameters were evaluated: hardness, adhesiveness, springiness, cohesiveness, gumminess, chewiness, and resilience. Hardness, defined as the maximum force required to deform the sample, showed significant variation between samples (Table 9). The highest value was recorded for fresh beetroot (S2; 302.2 ± 40.1 N), while the lowest was found in the sample placed in a 50% inulin solution (S3; 83.8 ± 8.8 N) and in water without additives (S1; 87.0 ± 19.0 N). These results indicate that the soaking/dehydration process significantly reduces the hardness of plant tissues, probably as a result of osmotic diffusion of water and solution components into or out of the cells. Gumminess, which is the product of hardness and cohesiveness, reflects the force required to break down a product ready for ingestion. The highest level of gumminess was observed in sample S2 (18,738 ± 2386), confirming that fresh beetroot is structurally the most compact. In the case of sample S6, placed in an inulin solution with magnesium chloride, gumminess was the highest among the samples treated with solutions (5358 ± 924), suggesting possible interactions between Mg^2+^ ions and inulin polysaccharides, leading to the strengthening of the tissue matrix. Chewiness, defined as the product of gumminess and springiness, provides information about the total effort required to chew a product. Fresh beetroot (S2) showed the highest chewiness (11,751 ± 1833), while significantly lower values were obtained in samples placed in inulin (S3; 1781 ± 655) and erythritol solution (S4; 1435 ± 408). Reduced chewiness may be beneficial from the consumer’s point of view, especially for people who prefer products that are easier to chew or those with dental problems. Springiness, defined as the ability of a sample to return to its original shape after initial deformation, also changed under the influence of the solutions used. The highest value was observed in sample S2 (0.52 ± 0.05), while the lowest was observed in sample S4 (0.31 ± 0.04), placed in the erythritol solution. The observed differences may result from the plasticizing effect of sugar alcohols, which reduce the mechanical elasticity of tissues by increasing their hydration. The other parameters (adhesiveness, cohesiveness, and resilience) showed less variation and were not sufficiently sensitive to accurately reflect the differences in the structure of the analyzed samples.

## 3. Materials and Methods

### 3.1. Sample Collection

Beet (*Beta vulgaris* L.) was used as the material for the study. The beets were purchased from the Organic Farming Cooperative “Dolina Mogilnicy”. Immediately after purchase, the beets were peeled and cut into 1.0 cm × 1.0 cm × 1.0 cm cubes. Then, portions of 50 ± 0.5 g were weighed and placed in separate containers. The samples were frozen at −80 °C, where they were stored until osmotic dehydration and further tests were carried out.

### 3.2. Chemicals and Reagents

2,2-azino-bis-(3-ethyl-benzthiazoline-6-sulfonic acid), 2,2′-azo-bis-2-amidinopropane dihydrochloride, 2,2-diphenyl-1-picrylhydrazyl, fluorescein, (±)-6-hydroxy-2,5,7,8-tetramethylchromane-2-carboxylic acid, potassium persulfate, dipotassium hydrogen phosphate anhydrous, sodium dihydrogen phosphate, methyl alcohol, nitric acid, and lanthanum chloride were purchased from Sigma-Merck (Poznan, Poland). Osmotically active compounds (inulin HSI, xylitol, erythritol) were purchased from HORTIMEX Sp. z o.o (Konin, Poland). Magnesium citrate (MyVita, Legnica Poland), magnesium oxide, magnesium chloride (NOW Food’s, Goczałkowice-Zdrój, Poland), and sucrose were purchased from an online store (Carrefour, Poznań, Poland). The remaining reagents used for the analyses were purchased from AlfaChem (Lublin, Poland).

### 3.3. Osmotic Dehydration Procedure

The impact of different osmotic dehydration conditions on the magnesium content of the tested samples was evaluated in two stages. The first stage examined the effect of the chemical form of magnesium (magnesium oxide, magnesium chloride, magnesium citrate), the type of osmotically active substance (inulin, erythritol, xylitol, sucrose), and the concentration of osmotically active solutions (25% and 50%) on the magnesium content in beet. Among the various chemical forms of magnesium available on the market, those approved for use in food (Regulation No. 1925/2006 of the European Parliament and of the Council) were selected, which differ in terms of their form (organic/inorganic), water solubility, and elemental magnesium content.

The samples were prepared for osmotic dehydration as follows: (1) 62.5 g osmotically active substance (inulin, erythritol, xylitol or sucrose) and 187.5 g water or 125 g osmotically active substance and 125 g water were weighed to prepare solutions (25% and 50%), (2) 12.5 g of magnesium compounds were added to glass vessels containing the osmotically active substance solution (5.0% of the solution’s mass), (3) the magnesium was mixed and dissolved well, (4) then, 50 g of cubes of beet were added to the prepared solutions (ratio of beets to solution weight was 1:5), (5) the glass vessels were closed with a cap and placed in a shaking water bath (SWB 22N)—the dehydration process was carried out under the following conditions: 50 °C/120 min, (6) after 2 h, the dehydrated beets were drained on a sieve, then weighed and placed in a freezer (−80 °C/24 h), (7) then, the beets were lyophilized, weighed and ground into powder using a grinder (Bosch, Gerlingen, Germany). The prepared samples were used to determine the magnesium content.

Based on the results obtained in the first stage of the research, the second stage of the research involved the selection of samples dehydrated using 50% solutions of inulin and erythritol with the addition of magnesium chloride. In addition to the fact that beetroot dehydrated in inulin and erythritol solutions had the highest magnesium content, it should be noted that both inulin and erythritol have health benefits, as indicated in the Section 1.

The second stage of osmotic dehydration consisted of evaluating the remaining process conditions (time, temperature, level of magnesium compound addition) on the magnesium content in the dehydrated material. Osmotic dehydration was carried out in three time variants (60, 120, 180 min) and two temperature variants (30 and 50 °C) with the addition of 2.5 or 5.0% magnesium compounds. The samples prepared in this manner were used for further analyses.

### 3.4. Determination of Magnesium Content

One gram of the osmotically dehydrated sample was weighed into quartz crucibles and placed in a muffle furnace (450 °C) for complete mineralization. The samples were then dissolved with nitric acid (1 mol/L) (Suprapure, Merck, Rahway, NJ, USA). The magnesium content was analyzed in three replicates. The mineral solutions were measured by flame atomic absorption spectrometry (ZA3000; Hitachi, Tokyo, Japan) [61]. Analysis of the samples was conducted in triplicate. The magnesium absorption level is the ratio of magnesium content in dehydrated beet to the content of magnesium ions added to the hypertonic solution.

### 3.5. Extract Preparation for Antioxidant Activity Analysis

To determine the antioxidant activity of dehydrated beet, sample extraction was carried out. A 5 g amount of the sample was weighed into conical flasks, and 50 mL of a 50% methanol–water solution was added. The samples prepared were placed in a shaking water bath (SWB 22N, LaboPLAY, Bytom, Poland) for 120 min at 50 °C. Then, the samples were centrifuged (1500 rpm, 10 min), filtered, and used for further analysis. Extraction conditions were based on previous studies [62].

### 3.6. Antioxidant Activity Analysis

The antioxidant activity of the osmotically dehydrated samples was analyzed in the following tests: oxygen radical absorbance capacity (ORAC) assay, DPPH radical scavenging assay, and ABTS radical scavenging assay. Analysis of the samples was conducted in triplicate.

The oxygen radical absorbance capacity of the extracts was determined using the method developed by Ou et al. (2002) [63]. The magnesium-enriched beetroot extract was dissolved in phosphate buffer (pH 7.4). Then, a reaction mixture was prepared (0.04 µM disodium fluorescein and 0.075 M phosphate buffer). A solution of 153 nM AAPH was used as a source of peroxide radicals. The fluorescence of the samples was measured using a spectrofluorometer (Hitachi F-2700, Hitachi, Tokyo, Japan) at an excitation wavelength of λ = 493 nm and an emission wavelength of λ = 515 nm. The results were expressed as µM Trolox equivalent per 1 g dry sample weight.

The determination of antioxidant activity using DPPH radicals was carried out according to the method developed by Brand-Williams et al. (1995) [64]. A DPPH reagent was prepared by weighing 0.01 g of DPPH and transferring it to a volumetric flask (25 mL) with a solvent (80% methanol-water solution). The procedure for determining the antioxidant activity was as follows: 100 µL of dehydrated beet extract was taken, 250 µL of DPPH reagent, and 2.0 mL of methanol-water solution were added. The reaction mixture was shaken in a vortex at room temperature (22 °C) and left to stand in the dark for 20 min. The absorbance of the samples was measured at a wavelength of λ = 517 nm using a spectrophotometer (Metertech SP 830, NanGang, Taipei, Taiwan). The result was expressed as mg Trolox/100 g dry weight of the sample.

The ability of magnesium-enriched beet extracts to reduce ABTS•+ cation radicals was performed according to the method described by Re et al. (1999) [65]. An ABTS solution was prepared by weighing 0.192 g of ABTS into a volumetric flask (50 mL) and filling it up with deionized water. Then, the potassium peroxodisulfate solution was prepared by weighing it (0.0166 g), transferring it to a volumetric flask (25 mL), and adding deionized water. Then, the ABTS and potassium peroxodisulfate solutions were combined in a ratio of 1.0:0.5 *v*/*v*. The mixture was stored at room temperature (22 °C) in the dark for 16 h. The procedure for determining the antioxidant activity was as follows: 30 µL of extract was taken, and 3 mL of ABTS reagent was added and shaken with a vortex mixer. After 6 min, the absorbance (λ = 734 nm) was measured using a spectrophotometer (Metertech SP 830). The result was expressed as mg Trolox/100 g dry weight of the sample.

### 3.7. Food Composition Analysis

The protein, fat, and fiber content were determined in the dehydrated beet. For the determination of protein content, The Kjeldahl method with the use of Kjeltec-2200 System (Foss Tecator, Hoganas, Sweden) was used according to AOAC [66]. For lipid extraction Soxhlet method using the Soxtec-HT6 System (Foss Tecator, Hoganas, Sweden) was used [67]. Total dietary fiber content was determined using the enzymatic-gravimetric Asp method using Fibertec (Foss Tecator, Sweden) [68]. Analysis of the samples was conducted in triplicate.

### 3.8. Texture Profile Analysis (TPA)

Texture profile analysis (TPA) was performed using TA.XTplus Texture Analyzer (Stable Micro Systems Ltd., Godalming, UK) with texturograph software (version 7.0.6.0 Stable Microsystems) according to Kowalczewski et al., 2019 [69].

### 3.9. Statistical Analysis

Statistically significant differences were determined using multivariate analysis of variance (ANOVA), followed by Tukey post hoc test (Statistica 13.1, StatSoft Inc., Kraków, Poland). The differences were considered statistically significant at *p* < 0.05. The experiments were conducted in triplicate.

## 4. Conclusions

Our research has confirmed that osmotic dehydration is an effective method of modifying the nutritional value of the plant matrix. In this experiment, it was proven that the effectiveness of osmotic dehydration is determined by the process conditions, with factors such as the chemical form and concentration of magnesium, the type of osmotic active substance, and time having the greatest impact on the result. The highest magnesium content was found in samples dehydrated under the following conditions: 50% erythritol solution, 5.0% magnesium chloride concentration, 180 min, 50 °C (678.91 mg/100 g) and 50% inulin solution, 5.0% magnesium chloride concentration, 180 min, 30 °C (672.45 mg/100 g).

However, it should be noted that osmotic dehydration can be a disadvantageous process as it can reduce the antioxidant activity of the tested samples. This can be prevented by adding ingredients with a high antioxidant potential (e.g., herbs, spices) to the hypertonic solution. Furthermore, it is possible to choose the proper ingredients for preparing hypertonic solutions. Instead of using sugar and polyol solutions, which have marginal antioxidant activity, it is possible to use vegetable or fruit juices and concentrates, for example.

At the same time, it should be noted that, depending on the type of hypertonic solution used and the type of chemical form of the bioactive substance used, the sensory characteristics of the dehydrated food may be altered. Therefore, it is also important to consider consumer acceptance of food products as part of the research process when designing food products using osmotic dehydration.

## Figures and Tables

**Table 1:** Magnesium content of dehydrated beet (mg/100 g) before freeze-drying (stage 1).

Osmotically Active Substance	Magnesium Oxide	Magnesium Citrate	Magnesium Chloride
Water	105.40 ± 2.56 ^a^A	87.31 ± 7.23 ^a^B	144.81 ± 11.89 ^a^C
IS25	303.25 ± 7.71 ^b^A	335.75 ± 6.92 ^b^B	399.37 ± 5.03 ^b^C
IS50	520.41 ± 11.23 ^c^A	577.10 ± 7.51 ^c^B	683.96 ± 10.77 ^c^C
XS25	255.90 ± 4.76 ^d^A	309.96 ± 12.76 ^d^B	327.36 ± 8.77 ^d^C
XS50	470.75 ± 7.52 ^e^A	569.82 ± 6.39 ^c^B	604.78 ± 10.74 ^e^C
ES25	401.72 ± 10.82 ^f^A	499.95 ± 7.63 ^e^B	479.58 ± 9.69 ^f^C
ES50	585.52 ± 9.51 ^g^A	612.05 ± 5.15 ^f^B	668.38 ± 5.67 ^c^C
SS25	282.19 ± 8.91 ^b^A	456.41 ± 9.61 ^g^B	475.87 ± 4.55 ^f^C
SS50	477.99 ± 10.33 ^e^A	497.62 ± 11.44 ^e^B	589.81 ± 7.08 ^e^C

^a,b,c,d,e,f,g^—different letters mean statistically significant differences (*p* < 0.05) between the effect of type of osmotically active substance on the magnesium content in beet samples; A,B,C—different letters mean statistically significant differences (*p* < 0.05) between the effect of chemical forms on the magnesium content in beet samples; values are means of three determinations ± SD. IS25 and IS50—inulin solution (25 and 50%); XS25 and XS50—xylitol solution (25 and 50%); ES25 and ES50—erythritol solution (25 and 50%); SS25 and SS50—sucrose solution (25 and 50%).

**Table 2:** Results of multivariate analysis of variance for dehydrated beetroot samples (stage 1).

Combinations of Factors	*F*	*df*	*p*	*η* ^2^
Chemical form of magnesium	1062.69	2.54	0.000	0.98
Osmotically active substance	3225.46	8.54	0.000	1.00
Chemical form of magnesium × Osmotically active substance	47.93	16.54	0.000	0.93

F—statistics analysis of variance, df—degrees of freedom, *p*—level of statistical significance, η^2^—effect strength.

**Table 3:** Magnesium content of dehydrated beet (mg/100 g) after freeze-drying (stage 1).

Osmotically Active Substance	Magnesium Oxide	Magnesium Citrate	Magnesium Chloride
Water	1803.54 ± 43.76 ^a^A	1658.47 ± 137.32 ^a^B	2030.05 ± 166.69 ^a^C
IS25	1609.32 ± 75.04 ^ab^A	1128.28 ± 51.04 ^b^B	1234.1 ± 75.43 ^b^C
IS50	1269.33 ± 71.11 ^e^A	1635.79 ± 24.26 ^a^B	1820.78 ± 10.89 ^c^C
XS25	979.22 ± 49.32 ^c^A	867.89 ± 35.71 ^c^B	1006.71 ± 129.07 ^d^A
XS50	976.9 ± 43.83 ^c^A	931.08 ± 29.03 ^cd^B	1043.34 ± 17.65 ^bd^C
ES25	1670.03 ± 83.83 ^ad^A	1379.47 ± 39.32 ^e^B	1585.52 ± 37.53 ^e^C
ES50	1508.8 ± 56.03 ^bd^A	1273.69 ± 80.01 ^be^B	1821.89 ± 55.88 ^c^C
SS25	1912.16 ± 76.26 ^a^A	1693.3 ± 35.66 ^a^B	1056.93 ± 58.66 ^bd^C
SS50	2260.42 ± 95.56 ^f^A	1077.65 ± 17.21 ^bd^B	1201.22 ± 14.42 ^b^C

^a,b,c,d,e,f^—different letters mean statistically significant differences (*p* < 0.05) between the effect of type of osmotically active substance on the magnesium content in beet samples; A, B, C—different letters mean statistically significant differences (*p* < 0.05) between the effect of chemical forms on the magnesium content in beet samples; values are means of three determinations ± SD. IS25 and IS50—inulin solution (25 and 50%); XS25 and XS50—xylitol solution (25 and 50%); ES25 and ES50—erythritol solution (25 and 50%); SS25 and SS50—sucrose solution (25 and 50%).

**Table 4:** Magnesium content of dehydrated beet (mg/100 g) before freeze-drying (stage 2).

Time/Temperature		IS50		ES50	
		Magnesium Chloride 2.5%	Magnesium Chloride 5.0%	Magnesium Chloride 2.5%	Magnesium Chloride 5.0%
60 min	30 °C	194.93 ± 8.69 ^aAxX^	416.33 ± 4.82 ^aBxX^	156.57 ± 7.13 ^bAxX^	458.11 ± 10.34 ^bBxX^
	50 °C	181.60 ± 6.00 ^aAxY^	430.34 ± 9.91 ^aBxY^	178.85 ± 5.83 ^aAxY^	478.62 ± 7.13 ^bBxY^
120 min	30 °C	278.43 ± 5.32 ^aAyX^	641.40 ± 7.30 ^aByX^	321.71 ± 9.16 ^bAyX^	595.95 ± 7.38 ^bByX^
	50 °C	301.88 ± 4.47 ^aAyY^	664.59 ± 12.57 ^aByY^	339.12 ± 4.86 ^bAyY^	644.58 ± 8.37 ^bByY^
180 min	30 °C	319.10 ± 4.66 ^azxX^	672.45 ± 6.88 ^aBzX^	355.92 ± 10.68 ^bAzX^	663.31 ± 7.62 ^aBzX^
	50 °C	326.64 ± 7.5.0 ^aAzxX^	655.52 ± 7.51 ^aByY^	329.61 ± 7.78 ^bAyY^	678.91 ± 6.94 ^bBzY^

^a,b^—different letters mean statistically significant differences (*p* < 0.05) between the effect of IS50 and ES50 on the magnesium content in beet samples; ^A,B^—different letters mean statistically significant differences (*p* < 0.05) between the effect of magnesium chloride 2.5% and 5.0% on the magnesium content in beet samples; ^x,y,z^—different letters mean statistically significant differences (*p* < 0.05) between the effect of different time conditions (60, 120, 180 min) on the magnesium content in beet samples; ^X,Y^—different letters mean statistically significant differences (*p* < 0.05) between the effect of different temperature conditions (30 and 50 °C) on the magnesium content in beet samples. IS50—inulin solution (50%); ES—erythritol solution (50%).

**Table 5:** Results of multivariate analysis of variance for dehydrated beetroot samples (stage 2).

Combinations of Factors	*F*	*df*	*p*	*η* ^2^
Osmotically active substance	29.23	1.48	0.000	0.38
Magnesium concentration	28,965.34	1.48	0.000	1.00
Process time	4179.19	2.48	0.000	0.99
Process temperature	38.84	1.48	0.000	0.45
Osmotically active substance × Magnesium concentration	3.41	1.48	0.071	0.07
Osmotically active substance × Process time	2.83	2.48	0.069	0.11
Osmotically active substance × Process temperature	7.60	1.48	0.008	0.14
Magnesium concentration × Process time	133.63	2.48	0.000	0.85
Magnesium concentration × Process temperature	11.48	1.48	0.001	0.19
Process time × Process temperature	27.75	2.48	0.000	0.54
Osmotically active substance × Magnesium concentration × Process time	121.57	2.48	0.000	0.84
Osmotically active substance × Magnesium concentration × Process temperature	9.92	1.48	0.003	0.17
Osmotically active substance × Process time × Process temperature	2.97	2.48	0.061	0.11
Magnesium concentration × Process time × Process temperature	0.29	2.48	0.748	0.01
Osmotically active substance × Magnesium concentration × Process time × Process temperature	14.69	2.48	0.000	0.38

F—statistics analysis of variance, df—degrees of freedom, *p*—level of statistical significance, η^2^—effect strength.

**Table 6:** The level of magnesium absorption (%) in the analyzed samples of osmotic dehydrated beetroot.

Time and Temperature		IS50		ES50	
		Magnesium Chloride 2.5%	Magnesium Chloride 5.0%	Magnesium Chloride 2.5%	Magnesium Chloride 5.0%
60 min	30 °C	13.04 ± 0.58 ^aAxX^	13.92 ± 0.16 ^aBxX^	10.47 ± 0.48 ^bAxX^	15.32 ± 0.35 ^bBxX^
	50 °C	12.15 ± 0.40 ^aAxY^	14.39 ± 0.33 ^aBxX^	11.96 ± 0.39 ^aAxY^	16.01 ± 0.24 ^bBxY^
120 min	30 °C	18.62 ± 0.36 ^aAyX^	21.45 ± 0.24 ^aByX^	21.52 ± 0.61 ^bAyX^	19.93 ± 0.25 ^bByX^
	50 °C	20.19 ± 0.30 ^aAyY^	22.23 ± 0.42 ^aByY^	22.68 ± 0.33 ^bAyY^	21.56 ± 0.28 ^bByY^
180 min	30 °C	21.34 ± 0.31 ^aAzX^	22.49 ± 0.23 ^aBzX^	23.81 ± 0.71 ^bAzX^	22.18 ± 0.25 ^aBzX^
	50 °C	21.85 ± 0.50 ^aAzX^	21.92 ± 0.25 ^aByX^	22.05 ± 0.52 ^aAyY^	22.71 ± 0.23 ^bBzX^

^a,b^—different letters mean statistically significant differences (*p* < 0.05) between the effect of IS50 and ES50 on the magnesium content in beet samples; ^A,B^—between the effect of magnesium chloride 2.5% and 5.0% on the magnesium content in beet samples; ^x,y,z^—different letters mean statistically significant differences (*p* < 0.05) between the effect of different time conditions (60, 120, 180 min) on the magnesium content in beet samples; ^X,Y^—different letters mean statistically significant differences (*p* < 0.05) between the effect of different temperature conditions (30 and 50 °C) on the magnesium content in beet samples. IS50—inulin solution (50%); ES—erythritol solution (50%).

**Table 7:** Magnesium content of dehydrated beet (mg/100 g) after freeze-drying (stage 2).

Time and Temperature		IS50		ES50	
		Magnesium Chloride 2.5%	Magnesium Chloride 5.0%	Magnesium Chloride 2.5%	Magnesium Chloride 5.0%
60 min	30 °C	479.53 ± 21.39 ^aAxX^	1265.63 ± 14.67 ^aAxX^	288.09 ± 13.12 ^bAxyX^	875 ± 19.75 ^bBxX^
	50 °C	473.99 ± 15.66 ^aAxX^	1170.52 ± 26.97 ^aBxY^	284.37 ± 9.27 ^bAxX^	1033.83 ± 15.4 ^bBxY^
120 min	30 °C	643.18 ± 12.29 ^aAyX^	1590.68 ± 18.1 ^aByX^	692.65 ± 19.72 ^bAyX^	1358.76 ± 16.83 ^bByX^
	50 °C	646.03 ± 9.56 ^aAyX^	1322.54 ± 25.02 ^aByY^	586.68 ± 8.41 ^bAyY^	1379.41 ± 17.91 ^bByX^
180 min	30 °C	650.96 ± 9.51 ^aAyX^	1519.75 ± 15.54 ^aBzX^	722.51 ± 21.67 ^bAyX^	1386.31 ± 15.93 ^bByX^
	50 °C	604.29 ± 13.87 ^aAzY^	1586.36 ± 18.18 ^aBzY^	547.15 ± 12.92 ^bAzY^	1167.73 ± 11.93 ^bBzY^

^a,b^—different letters mean statistically significant differences (*p* < 0.05) between the effect of IS50 and ES50 on the magnesium content in beet samples; ^A,B^—different letters mean statistically significant differences (*p* < 0.05) between the effect of magnesium chloride 2.5% and 5.0% on the magnesium content in beet samples; ^x,y,z^—different letters mean statistically significant differences (*p* < 0.05) between the effect of different time conditions (60, 120, 180 min) on the magnesium content in beet samples; ^X,Y^—different letters mean statistically significant differences (*p* < 0.05) between the effect of different temperature conditions (30 and 50 °C) on the magnesium content in beet samples. IS50—inulin solution (50%); ES—erythritol solution (50%).

**Table 8:** Proximate composition of osmotic dehydrated beetroot.

	Protein Content (g/100 g)	Fat Content (g/100 g)	Dietary Fiber Content (g/100 g)
A1	1.21 ± 0.15 ^a^	0.09 ± 0.01 ^a^	1.57 ± 0.13 ^a^
A2	6.34 ± 0.15 ^b^	0.55 ± 0.04 ^b^	5.81 ± 0.18 ^c^
B1	1.65 ± 0.07 ^a^	0.10 ± 0.02 ^a^	1.51 ± 0.09 ^a^
B2	8.06 ± 0.28 ^b^	0.79 ± 0.04 ^b^	6.26 ± 0.28 ^b^
C1	1.43 ± 0.05 ^a^	0.15 ± 0.02 ^a^	1.44 ± 0.07 ^a^
C2	7.34 ± 0.14 ^b^	0.79 ± 0.03 ^b^	6.38 ± 0.10 ^b^
D1	1.65 ± 0.04 ^a^	0.16 ± 0.01 ^a^	1.67 ± 0.14 ^a^
D2	8.27 ± 0.12 ^b^	0.83 ± 0.04 ^b^	6.84 ± 0.16 ^b^
E1	2.14 ± 0.18 ^a^	0.42 ± 0.02 ^a^	2.15 ± 0.13 ^b^
E2	9.56 ± 0.41 ^c^	1.70 ± 0.08 ^c^	13.01 ± 0.36 ^c^

^a,b,c^—different letters mean statistically significant differences (*p* < 0.05). Sample A1/A2—beet subjected to osmotic dehydration (50% inulin solution, 5.0% potassium chloride addition, 180 min, 30 °C, before and after freeze-drying; Sample B1/B2—beet subjected to osmotic dehydration (50% inulin solution, 5.0% potassium chloride addition, 180 min, 50 °C, before and after freeze-drying; Sample C1/C2—osmotically dehydrated beet (50% erythritol solution, 5.0% potassium chloride addition, 180 min, 30 °C, before and after freeze-drying; Sample D1/D2—osmotically dehydrated beet (50% inulin solution, 5.0% potassium chloride addition, 180 min, 50 °C, before and after freeze-drying; Sample E1—fresh beet; Sample E2—freeze-dried beet.

**Table 9:** Texture profile analysis of beetroot (TPA).

	Hardness (N)	Adhesiveness (N × s)	Springiness (%)	Cohesiveness	Gumminess	Chewiness	Resilience
S1	87.0 ± 19.0	−14.40 ± 6.67	0.41 ± 0.03	0.28 ± 0.09	2520 ± 468	1256 ± 149	0.19 ± 0.07
S2	302.2 ± 40.1	−14.01 ± 0.29	0.52 ± 0.05	0.46 ± 0.14	18,738 ± 2386	11,751 ± 1833	0.32 ± 0.12
S3	83.8 ± 8.8	−17.59 ± 1.57	0.46 ± 0.06	0.36 ± 0.10	3087 ± 627	1781 ± 655	0.25 ± 0.08
S4	104.1 ± 36.3	−16.80 ± 2.39	0.31 ± 0.04	0.31 ± 0.04	3121 ± 774	1435 ± 408	0.22 ± 0.04
S5	126.0 ± 22.4	−14.82 ± 9.18	0.44 ± 0.08	0.29 ± 0.04	3694 ± 1006	1844 ± 505	0.20 ± 0.03
S6	147.2 ± 52.9	−16.59 ± 7.04	0.48 ± 0.05	0.40 ± 0.08	5358 ± 924	2380 ± 327	0.29 ± 0.06
S7	95.9 ± 19.6	−17.66 ± 5.04	0.40 ± 0.02	0.37 ± 0.10	3109 ± 1114	1706 ± 600	0.26 ± 0.07

S1—fresh beet placed in water; S2—fresh beet; S3—beet subjected to osmotic dehydration (50% inulin solution, without potassium chloride); S4—beet subjected to osmotic dehydration (50% erythritol solution, without potassium chloride); S5—beet placed in a 5.0% magnesium chloride solution; S6—beet subjected to osmotic dehydration (50% inulin solution, 5.0% magnesium chloride); S7—beet subjected to osmotic dehydration (50% erythritol solution, 5.0% magnesium chloride).

## Data Availability

The data presented in this study are available on request from the corresponding author.
